# Altitudinal Patterns of Leaf Traits and Leaf Allometry in Bamboo *Pleioblastus amarus*

**DOI:** 10.3389/fpls.2018.01110

**Published:** 2018-07-31

**Authors:** Ziwu Guo, Hua Lin, Shuanglin Chen, Qingping Yang

**Affiliations:** ^1^Research Institute of Subtropical Forestry, Chinese Academy of Forestry, Hangzhou, China; ^2^Forestry Bureau of Shaxian County, Shaxian, China

**Keywords:** bamboos, altitude, leaf functional traits, clonal plants, allometric growth relationships

## Abstract

Awareness of local-scale variation in leaf traits for a single species and the relationships between these traits and their dependence on altitude might be essential for extrapolating ecophysiological processes from the leaf to the ecosystem level. While altitudinal patterns of leaf traits have been extensively studied in a number of species, little is known about such patterns in bamboos. We analyzed leaf functional traits and leaf allometric relationships of *Pleioblastus amarus* at three different altitudes (200, 400, and 800 m). With increasing altitude, most functional traits, including leaf length, width, perimeter, area, dry weight, and water content, decreased significantly, while the leaf length:width ratio exhibited a marked increase, resulting in a tendency toward narrow leaves. Specific leaf area first increased, and then decreased, while the change in leaf dry matter content showed the opposite trend. Leaf area was positively correlated with leaf length, leaf width and leaf perimeter, but negatively correlated with the leaf length:width ratio. With increasing altitude, the slopes of these relationships for leaf area first increased, and then decreased. Leaf biomass was positively correlated with leaf length, width, perimeter, and area, with the slopes of the relationships being the same at all altitudes. Thus, the leaves of this bamboo species at middle altitude have the highest specific leaf area and lowest leaf dry matter content. Our findings suggest that this bamboo species has a big potential of growth and morphological plasticity.

## Introduction

Leaves are the most important component of a plant’s photosynthetic apparatus and play a pivotal role in plant function and long-term adaptation to environmental change (Liu Z.L. et al., 2016). Leaf morphology, biomass, and water content are fundamental leaf functional traits (Wright et al., 2005). Specific leaf area and leaf dry matter content represent the balance between investing in growth or storage and are traits that have been widely used to predict growth strategy and the response to environmental variations. Therefore, studying leaf functional traits is important for scaling up to predict community- or ecosystem-level functions and processes (Scheepens et al., 2010; Daneshgar et al., 2012).

Allometry refers to the numerical relationship between growth and allocation of an organism, morphological variability can be understood as a change in a plant’s allometric trajectory. Allometric relationships exist widely from individuals to ecosystems, can be used to predict plant growth and ecosystem functions (e.g., productivity), and also embody the relationship with environmental changes (Chave et al., 2014; Paul et al., 2016). Allometric relationships among leaf traits, i.e., relative changes in leaf size, shape, and biomass, are both a cause and a consequence of variation in resource availability and environmental changes (Forrester et al., 2017; Zhang et al., 2017).

Environmental factors, such as air temperature, radiation, and soil nutrients, may covary with altitude (Körner, 2007; Soethe et al., 2008). For instance, temperature will decrease, while precipitation and radiation will increase with a rise in altitude (Read et al., 2014; Li et al., 2018; Wang et al., 2018). Such variations might influence leaf traits and their allometric relationships, as leaf traits may exhibit considerable plasticity in response to environmental demands (Valladares et al., 2007; Li and Yu, 2009; Zdravko, 2011). Altitudinal gradients can be used as model templates for large-scale studies that analyze the adaptive features of terrestrial plants under the influence of global climate change (Li et al., 2018; Wang et al., 2018). Awareness of local-scale variation in many leaf traits for individual species, as well as the relationships among these traits and their dependence on altitude, might be essential for extrapolating ecophysiological processes from the leaf to the ecosystem level (Navas et al., 2010).

Bamboos are forest species that have widespread distribution in the tropical and subtropical regions of the world (Zhang et al., 2014), with most bamboo species growing naturally in mountain areas along an altitudinal gradient. Their unique growth characteristics, including fast growth, high biomass production, and rapid maturation from shoot to culm, have enabled bamboos to become globally important resource of biomass, food, and timber (Nath et al., 2015). In comparison to most trees (10–50 years for harvest), the harvest time for bamboos is only 3–5 years (Desalegn and Tadesse, 2014). Furthermore, bamboos are typical clonal plants that can produce genetically identical modules (ramets) that are both physically and physiologically dependent on their connected parent ramets, at least at their earlier stages of development (Yu et al., 2008, 2009; Song et al., 2013; Wang et al., 2017). These ramets are formed by vegetative propagation and stay connected to the parent organs at least until they are established, thus forming sets of connected ramets, or clonal fragments (Dong et al., 2015; Guo et al., 2017; Wang et al., 2017). Elongation of bamboo rhizomes and sprouting of shoot buds result in widespread pure bamboo forests along the slopes of mountains. Natural bamboo forest is thus a result of ramets connected by rhizomes, and such forests along the slopes of mountains may have originated from only one or a few genets. However, few studies have examined the leaf traits of bamboos along an altitudinal gradient.

*Pleioblastus amarus* is an economically important bamboo species used for both timber and young shoots. Through rapid clonal growth via rhizomes, one genet of this species can produce numerous ramets (stems) that spread over a large distance so that genetically uniform patches of ramets can be formed (Dong, 2011). In this study, the leaf shape traits and biomass of *P. amarus* were determined at three altitudes (200, 400, and 800 m a.s.l.) of Xuefeng Mountain in China. Based on these data, we analyzed the variation in leaf traits and their relationships along an altitudinal gradient at the individual species level and addressed the following questions: (1) How do the leaf traits change along the altitudinal gradient? (2) Do variations in leaf shape and biomass result in corresponding differences in the allometric relationships of leaf traits among the altitudes? By addressing these questions, we were able to infer the altitude at which this bamboo species can achieve better performance.

## Materials and Methods

### Study Area

Xuefeng Mountain (26°29–26°40′N, 117°32–117°46E) is the highest mountain of the Shaxian in Fujiang Province, China. The altitude of this mountain ranges from 100 to 1298 m, and the forest soil is classified as “red soil.” The vegetation varies with elevation: at the bottom of the mountain, below 200 m, most of the area is cropland; at the middle range, from 200 to 800 m, *P. amarus* forest is the main vegetation type; and above this elevation, evergreen broadleaf forest is widespread and universal.

### Study Design and Sampling

Along a transect of the southern slope of Xuefeng Mountain, three altitudinal zones were investigated, referred to here as low (200 ± 20 m), middle (400 ± 20 m), and high (800 ± 20 m) altitudinal zones. Six experimental plots (10 m × 10 m) were set in each altitudinal zone. The forest structure of each altitudinal zone was investigated and is summarized in **Table 1**. In May 2016, nine bamboo plants for each age class (1-year-old, 2-year-old, and 3-year-old bamboos) were chosen for leaf sampling within each plot. Thirty fully expanded, mature, and healthy leaves from the upper, middle, and bottom crown of each selected plant were cut off with scissors, and 10 leaves were then randomly chosen per plant.

**Table 1:** Summary of *Pleioblastus amarus* forest structure.

Altitude	Density (stem hm^−2^)	Diameter at Breast height (cm)	Age Structure (1a:2a:3a)
Low altitude (200 m)	5230 ± 136	3.6 ± 0.27	3.42:4.13:2.35
Middle altitude (400 m)	4860 ± 113	4.3 ± 0.32	3.53:4.28:2.19
High altitude (800 m)	4430 ± 109	4.2 ± 0.31	3.31:4.13:2.46

The fresh mass (g) for each leaf was measured immediately with an SE202F Ohaus–CN electronic balance (Ohaus Corporation, United States). Leaf length (cm) and width (cm) were measured with rulers, and the length:width ratio was calculated. Leaf area (cm^2^ per individual) and perimeter (cm) were measured using a scanner (Cano Scan LIDE 110, Japan) and Photoshop CS (Adobe, United States). All leaves were then placed in a drying oven for a minimum of 72 h at 80°C, and the final dry mass (g) was measured with an electronic balance. Specific leaf area (SLA; cm^2^ g^−1^) was calculated as: SLA = leaf area/leaf dry weight. Leaf dry matter content (LDMC, g.g^−1^) was calculated and expressed as a ratio of dry weight to fresh weight.

### Data Analysis

The variation in leaf traits for *P. amarus* with altitude was tested with one-way ANOVA. Two-way ANOVA was used to investigate the effects of altitude and bamboo age on the leaf traits of *P. amarus*. All values presented are means ± SD.

The scaling relationship between leaf biomass (leaf dry weight) or leaf area and other leaf functional traits is described by the equation:

$$Y=a\times x^{b}\text{ or }\log(y)=\log(a)+b\times \log(x)$$

where *y* is leaf area or leaf biomass, and *x* refers to other leaf traits; *a* is the *y*-intercept and *b* is the slope of the scaling function, representing the allometric exponent.

To examine these scaling relationships at the three altitudes with different bamboo ages, we used model II regression (also known as reduced major axis, or RMA) on log-transformed values of leaf traits. If the slopes of these relationships were not significantly different from |1.00| (at the 95% confidence level), they were deemed to be isometric. If the slopes of these relationships were not significantly different among altitudes, then in addition to the original attitude-specific values of the slopes, the common standardized major axis (SMA) slope was reported, the latter in brackets. The SMA regression was performed to examine the scaling slope using the “smart” package. All statistics were analyzed using the *R* platform (R Development Core Team, 2013) and Microsoft Excel 2016.

## Results

### Leaf Functional Traits at Different Altitudes

**FIGURE 1:**
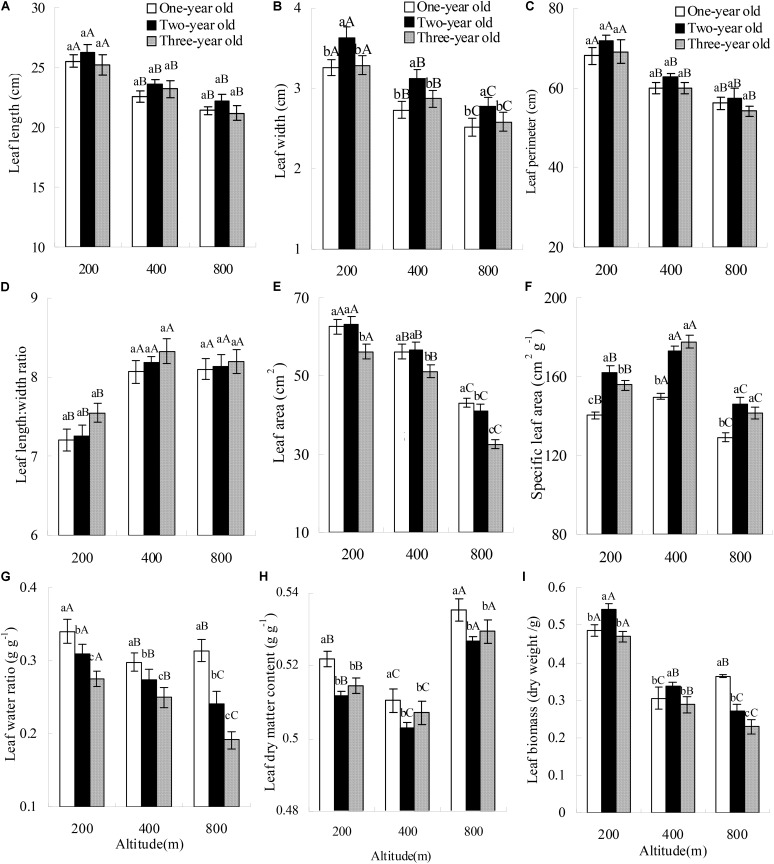
Leaf functional traits of *Pleioblastus amarus* at different altitudes. Different upper-case letters indicate significant (*P <* 0.05) differences among different altitudes with the same bamboo age class, while different lower-case letters indicate significant (*P <* 0.05) differences among different bamboo age classes at the same altitude.

Altitude and bamboo age significantly affected leaf width, leaf perimeter, leaf area, SLA, leaf water content, and leaf dry weight (**Table 2**). All these variables decreased markedly with increasing altitude (**Figures 1A–C,E,G,I**), except SLA (**Figure 1F**), which first increased and then exhibited a marked decrease. There was an obvious interaction effect of altitude and bamboo age on these variables. Altitude also affected leaf length, leaf length:width ratio and LDMC, whereas bamboo age had no effect on these variables. Furthermore, there was no interaction effect of altitude and bamboo age on these variables. With increasing altitude, leaf length decreased (**Figure 1A**), but the leaf length:width ratio increased greatly (**Figure 1D**), with leaves tending to narrow. LDMC decreased markedly and then increased with increasing altitude (**Figure 1H**).

**Table 2:** Analysis of variance results for the effect of altitude and bamboo age on leaf traits of *Pleioblastus amarus.*

Effect	*df*	Leaf length (cm)	Leaf width (cm)	Leaf length : width ratio	Leaf area (cm^2^)	Specific leaf area (cm^2^ g^−1^)	Leaf water ratio (g g^−1^)	Leaf dry weight (g)	Leaf dry matter content (g g^−1^)
Altitude	2	32.13^∗∗^	69.67^∗∗^	21.166^∗∗^	86.11^∗∗^	64.08^∗∗^	29.08^∗∗^	148.54^∗∗^	7.68^∗^
Bamboo age	2	2.98^ns^	5.26^∗^	0.53^ns^	5.25^∗^	12.82^∗∗^	5.15^∗^	10.84^∗^	0.19^ns^
Altitude × Age	4	2.55^ns^	5.17^∗^	0.69^ns^	7.51^∗^	5.41^∗^	6.19^∗^	6.87^∗∗^	0.74^ns^

*df, degrees of freedom; F-values and significance levels (^∗∗^P < 0.01, ^∗^P < 0.05, and ^ns^P ≥ 0.05) are given.*

### Allometry of Leaf Area at Different Altitudes

Leaf area was positively correlated with leaf length, width and perimeter, and negatively correlated with the leaf length:width ratio (*P <* 0.05, **Table 3**). The SMA slopes for all these relationships were statistically different from |1.00| (*P <* 0.05). The steepest slopes were those for the relationships of leaf area with length, perimeter, and length:width ratio at 400 m, while the slopes for each of these relationships were nearly the same at 200 m as those at 800 m (*P* > 0.05). There was no difference in the scaling slope of the relationship between leaf area and width at the three altitudes, all sharing the same SMA slope (1.61). With increasing altitude, the SMA slope (|*b*|) for the relationship of leaf area with leaf length, leaf length:width ratio, and perimeter increased, then decreased, with the biggest value being that for the middle altitude (400 m).

**Table 3:** Allometric relationships between leaf area and other leaf functional traits of *Pleioblastus amarus* at different altitudes.

*y*-variable	*x*-variable	Altitude	*n*	*R^2^*	SMA slope	LowCI	UppCI	Test 1 (*P*-value)
Leaf area	Leaf length	200	90	0.75	1.94 (1.98)	1.75	2.16	0.00
		400	90	0.79	2.32	2.11	2.55	0.00
		800	90	0.69	2.04 (1.98)	1.81	2.30	0.00
	Leaf width	200	90	0.67	1.57 (1.61)	1.39	1.77	0.00
		400	90	0.73	1.59 (1.61)	1.42	1.77	0.00
		800	90	0.62	1.68 (1.61)	1.47	1.92	0.00
	Leaf length : width ratio	200	90	0.11	−1.76 (−1.77)	−2.18	−1.43	0.00
		400	90	0.23	−2.05	−2.50	−1.67	0.00
		800	90	0.12	−1.78 (−1.77)	−2.20	−1.45	0.00
	Leaf perimeter	200	90	0.69	1.55 (1.55)	1.38	1.74	0.00
		400	90	0.49	2.07	1.78	2.41	0.00
		800	90	0.42	1.55 (1.55)	1.32	1.82	0.00

*n, sample size; R^2^, determination coefficient; SMA slope, scaling slope. All data were log10-transformed before analysis. Numbers in brackets indicate the common SMA slope for the different altitudes when there was no statistically significant difference in the estimated exact slope values between the two (or among the three) altitudes to be compared. LowCI and UppCI are lower and upper limits for the 95% confidence intervals, respectively. Test1 (P-value) indicates the statistical significance of the SMA slope value being different from 1.*

### Allometry of Leaf Biomass at Different Altitudes

Leaf biomass was positively correlated with leaf length, width, perimeter, area and water content, and negatively correlated with length:width ratio (*P <* 0.05, **Table 4**). Most of the scaling slopes were statistically different from |1.00| (*P <* 0.05, **Table 4**). The exceptions were the scaling slopes of the relationship between leaf biomass and water content, which were close to |1.00| (*P* > 0.05, **Table 4**). Altitude had no effect on the scaling slope of the relationship between leaf biomass and leaf area (1.19), between leaf biomass and width (1.92), between leaf biomass and length (2.46), or between leaf biomass and water content (0.97), with the three altitudes sharing a common slope. The SMA slope |b| of the relationship between leaf biomass and leaf perimeter, and the relationship between leaf biomass and leaf length:width ratio, were both at low (200 m) equal to the corresponding slopes at high altitude (800 m), but significantly lower than the slopes at the middle altitude (400 m).

**Table 4:** Allometric relationships between leaf biomass (dry weight) and other leaf functional traits of *Pleioblastus amarus* at different altitudes.

*y*-variable	*x*-variable	Altitude	*n*	*R^2^*	SMA slope	LowCI	UppCI	Test 1 (*P*-value)
Leaf biomass	Leaf length	200	90	0.76	2.36 (2.46)	2.12	2.62	0.00
(dry weight)		400	90	0.48	2.77 (2.46)	2.38	3.22	0.00
		800	90	0.49	2.38 (2.46)	2.04	2.77	0.00
	Leaf width	200	90	0.51	1.91 (1.92)	1.65	2.21	0.00
		400	90	0.56	1.97 (1.92)	1.65	2.18	0.00
		800	90	0.50	1.90 (1.92)	1.69	2.29	0.00
	Leaf	200	90	0.32	−2.14 (−2.12)	−2.65	−1.74	0.00
	length : width ratio	400	90	0.33	−2.44	−2.98	−2.01	0.00
		800	90	0.41	−2.08 (−2.12)	−2.57	−1.69	0.00
	Leaf perimeter	200	90	0.70	1.88 (1.86)	1.67	2.11	0.00
		400	90	0.39	2.47	2.10	2.91	0.00
		800	90	0.36	1.81 (1.86)	1.53	2.15	0.00
	Leaf area	200	90	0.86	1.19 (1.19)	1.12	1.31	0.00
		400	90	0.71	1.21 (1.19)	1.06	1.33	0.00
		800	90	0.76	1.16 (1.19)	1.05	1.29	0.00
	Leaf water ratio	200	90	0.83	0.92 (0.97)	0.87	1.03	0.16
		400	90	0.71	1.05 (0.97)	0.95	1.18	0.94
		800	90	0.48	0.99 (0.97)	0.89	1.15	0.34

*n, sample size; R^2^, determination coefficient; SMA slope, scaling slope. All data were log10-transformed before analysis. Numbers in brackets indicate the common SMA slope for the different altitudes when there was no statistically significant difference in the estimated exact slope values between the two (or among the three) altitudes to be compared. LowCI and UppCI are lower and upper limits for the 95% confidence intervals, respectively. Test1 (P-value) indicates the statistical significance of the SMA slope value being different from 1.*

## Discussion

In mountain habitats, there can be large variations in temperature, moisture, light, soil properties, and other abiotic factors along altitudinal gradients (Read et al., 2014; Li et al., 2018; Wang et al., 2018). All these changes can greatly affect leaf functional traits and ultimately may result in some obvious changes to plant growth and development as well as population expansion (Dong, 2011). In the present study, leaf functional traits varied greatly among the three altitudes and with different bamboo ages. Overall, most of the leaf traits, including leaf length, width, perimeter, area, biomass (dry weight), and water content, decreased significantly with increasing altitude. Furthermore, the leaf length:width ratio increased markedly, indicating that leaves tend to be narrower at higher altitude. All these changes in leaf functional traits reduced contact area with the environment, and enhanced the plant’s tolerance to the low temperatures and high levels of moisture and radiation characteristic of higher altitudes (Kofidis et al., 2007; Reich et al., 2014). With increasing altitude, LDMC decreased significantly, and then increased, while the opposite was true for SLA variation. The bamboo leaves at the middle altitude (400 m) had the lowest LDMC and highest SLA, indicating a strengthening of their ability to capture light and produce dry matter (Long et al., 2011; Zhang et al., 2015). Bamboos are clonal plants with strong horizontal clonal growth and expansion ability (Dong, 2011; Lieurance et al., 2018). *P. amarus* is one of the most important amphipodial bamboos, and the buds along rhizomes or at culm bases all have the potential to grow into new bamboo plants. Owing to their high plasticity, populations may be able to expand from middle to lower or higher altitudes where growth conditions are less optimal. With increasing altitude, environmental stresses such as high solar radiation, low temperatures, nutrient loss, and other abiotic pressures will increase, while photosynthetic ability and carbon assimilation will decrease to some extent, together resulting in an obvious decrease in leaf dry weight. *P. amarus* must therefore enlarge its leaf area per unit biomass, as well as its production ability, to maintain sufficiently high levels of photosynthetic activity and population stability. Furthermore, SLA is an integrative parameter of leaf area and leaf dry weight, and increasing SLA at middle altitude may be one of the strategies adopted by bamboos to adapt to changing environments by maximizing their photosynthetic rate. At low altitude, bamboos face strong pressure from human disturbance, and their stand density is also higher, together resulting in a reduced SLA (**Table 1**).

Many abiotic factors, such as temperature, moisture, light, and soil properties have the potential to affect allometric relationships among plant traits (Misle et al., 2013; Jorquera-Fontena et al., 2017). In this study, leaf area correlated positively (leaf length, width, and perimeter) or negatively (leaf length:width ratio) with other leaf morphological traits, and the slopes of all these relationships were significantly different from |1.00|. With increasing altitude, the slopes for the relationships of leaf area with length, perimeter, and length:width ratio first increased, and then decreased, while the slope for the relationship between leaf area and leaf width increased slightly. The slopes for the relationships of leaf area with leaf length, perimeter, and length:width ratio at the middle altitude (400 m) were 2.32, 2.05, and 2.07, respectively, which were all significantly greater than those at the lower or higher altitude. These results suggest that leaf morphological traits examined showed flexible allometric relationships, and changes in leaf length, perimeter, and length:width ratio may result in a larger change in leaf area at middle altitude than at low and upper altitudes (Niklas et al., 2007; Yao et al., 2013; Forrester et al., 2017).

In this study, leaf biomass correlated positively with leaf morphological traits, with most of the slopes differing from |1.00|, the relationship between leaf biomass and water content being the only exception (0.97). All slopes for the relationships between leaf biomass and leaf morphological traits among different altitudes varied slightly. Most of the slopes for the relationships between leaf biomass and morphological traits were nearly equal for the three altitudes; specifically, the three altitudes had a common slope except for the relationship between leaf biomass and leaf perimeter and between leaf biomass and leaf length:width ratio. This result indicates that the relationship between leaf dry weight and other leaf traits is stable at different altitudes, demonstrating a strong covariant ability (Chave et al., 2014; Zhang et al., 2015).

Since the leaf traits of bamboos along the altitudinal gradient varied greatly, the allometric relationships among these leaf morphological traits were found to be plastic, which illustrate that this bamboo is tolerant to biotic and abiotic stresses of different altitudes with strong ecological adaptability. Furthermore, the fact that LDMC was the lowest, and SLA was the highest at the middle altitude indicates that growth and population expansion will be strongest at this altitude. It is well known that bamboos are typical clonal plants, with many ramets or stems of different ages originating from the same parent bamboo group (Liu F.H. et al., 2016; Guo et al., 2017). Furthermore, rhizome elongation and the sprouting of rhizome buds into stems or ramets propel the expansion of bamboo forests (Li et al., 2015; Luo et al., 2016). Thus, while middle altitude is clearly favorable for the growth of *P. amarus*, bamboos have a strong ability to grow and expand in lower- or upper-altitude areas through clonal growth.

## Conclusion

Bamboos are typical clonal plants and widespread in mountain areas. In the present study, altitude was found to greatly affect leaf functional traits, and there was an interaction effect with bamboos age on most leaf traits. With increasing altitude, most functional traits decreased significantly, and leaves tended to be narrower. Leaf area correlated positively with leaf length, leaf width, and leaf perimeter, but negatively with the length:width ratio. Leaf biomass correlated positively with leaf length, leaf width, leaf perimeter, leaf area, but negatively with the length:width ratio. Furthermore, the leaves of bamboos at the middle altitude (400 m) had the highest SLA and lowest LMDC; and the SMA slopes were greater at this altitude than at the lower (200 m) and higher (800 m) altitudes. Our findings suggest that this bamboo species has a great potential of growth and morphological plasticity.

## Author Contributions

ZG and SC conceived and designed the investigation. ZG performed the data analysis and wrote the manuscript. HL and QY conducted the field work, sampling, and pre-treatment of the samples.

## Conflict of Interest Statement

The authors declare that the research was conducted in the absence of any commercial or financial relationships that could be construed as a potential conflict of interest.
